# Supplementary material for: Phytochemicals Recovery from Grape Pomace: Extraction Improvement and Chemometric Study
Source: Foods. 2023 Feb 24;12(5):959. doi: 10.3390/foods12050959 (PMC10001001; doi:10.3390/foods12050959)
Supplement: Supplementary file 1 [file foods-12-00959-s001.zip › foods-2191260-supplementary.pdf]

__Supplementary Materials__

Phytochemicals Recovery from Grape Pomace: Process Optimization and Chemometric Study

Maura Ferri, Vasco Lima, Alessandro Zappi, Ana Luísa Fernando, Dora Melucci, Annalisa Tassoni

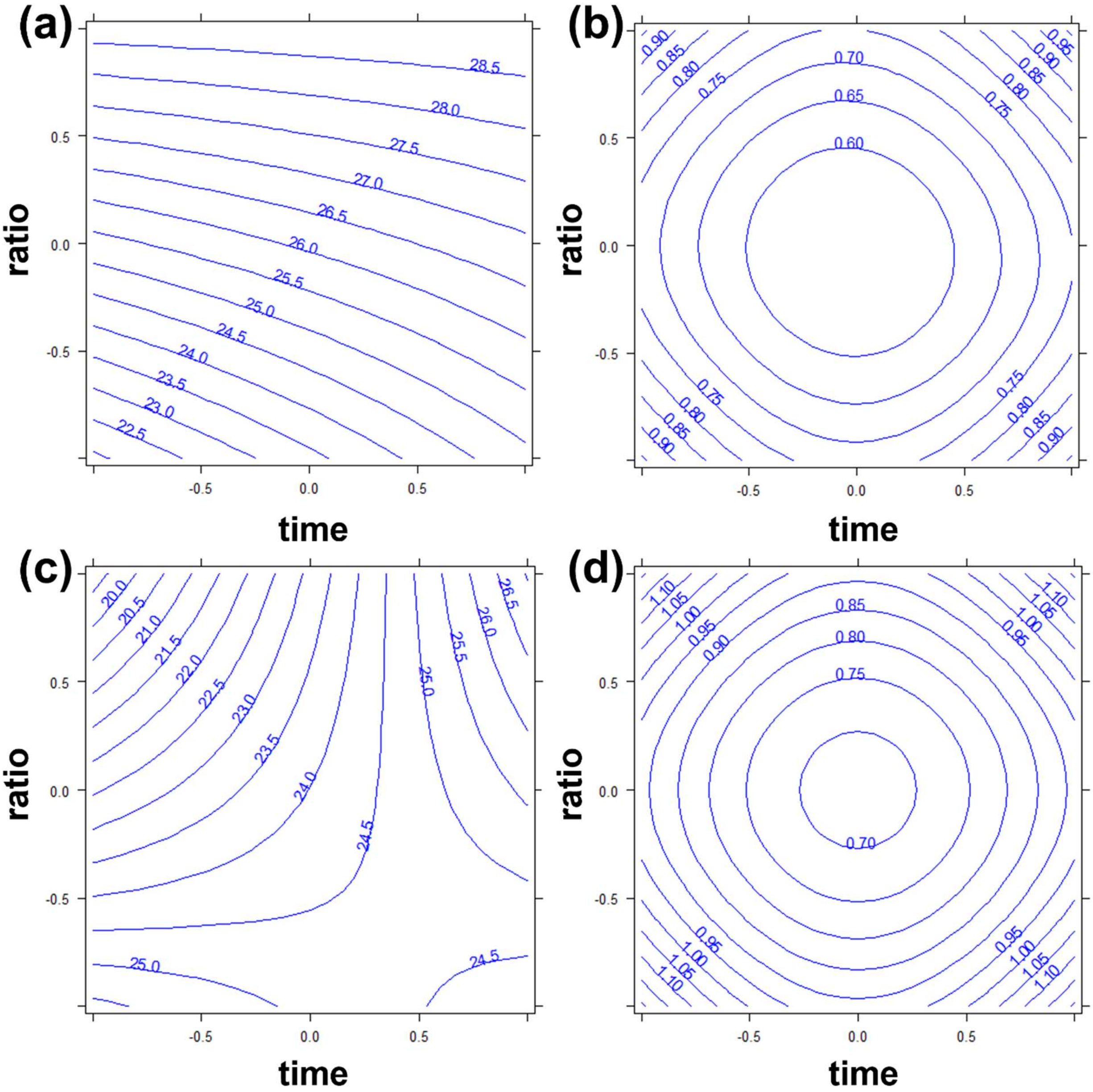

Supplementary Figure S1. (a,c) Response surface and (b,d) semiamplitude of the 95% confidence interval for (a,b) Merlot red GP and (c,d) white Garganega GP models. Factors time and ratio are reported in abscissa and ordinate respectively. Enzyme type was kept to Pectinex level. The axis scale ±1 is due to DoE levels.

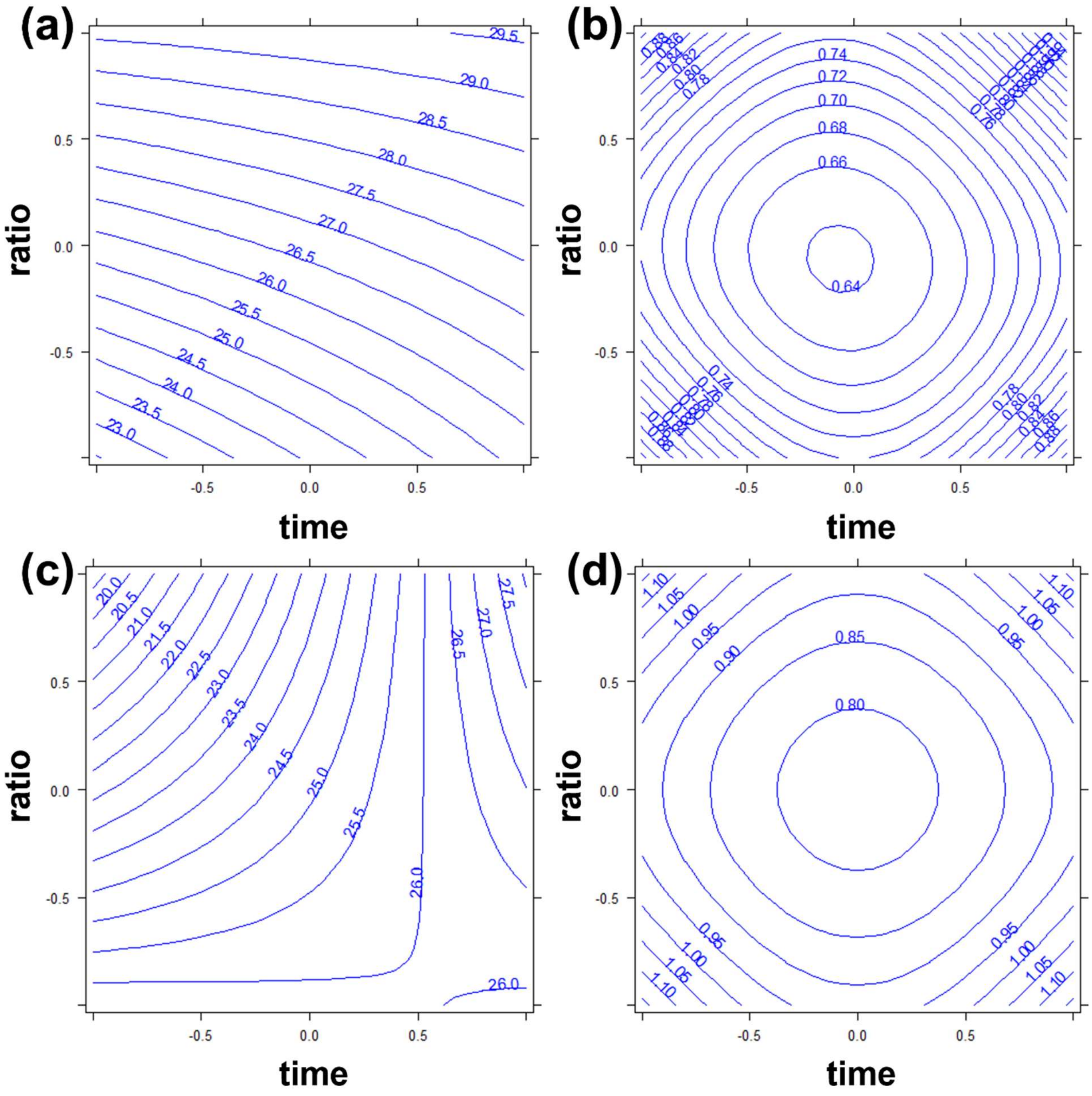

Supplementary Figure S2. (a,c) Response surface and (b,d) semiamplitude of the 95% confidence interval for (a,b) red Merlot GP and (c,d) white Garganega GP models. Factors time and ratio are reported in abscissa and ordinate respectively. Enzyme type was kept to Cellucast level. The axis scale ±1 is due to DoE levels.

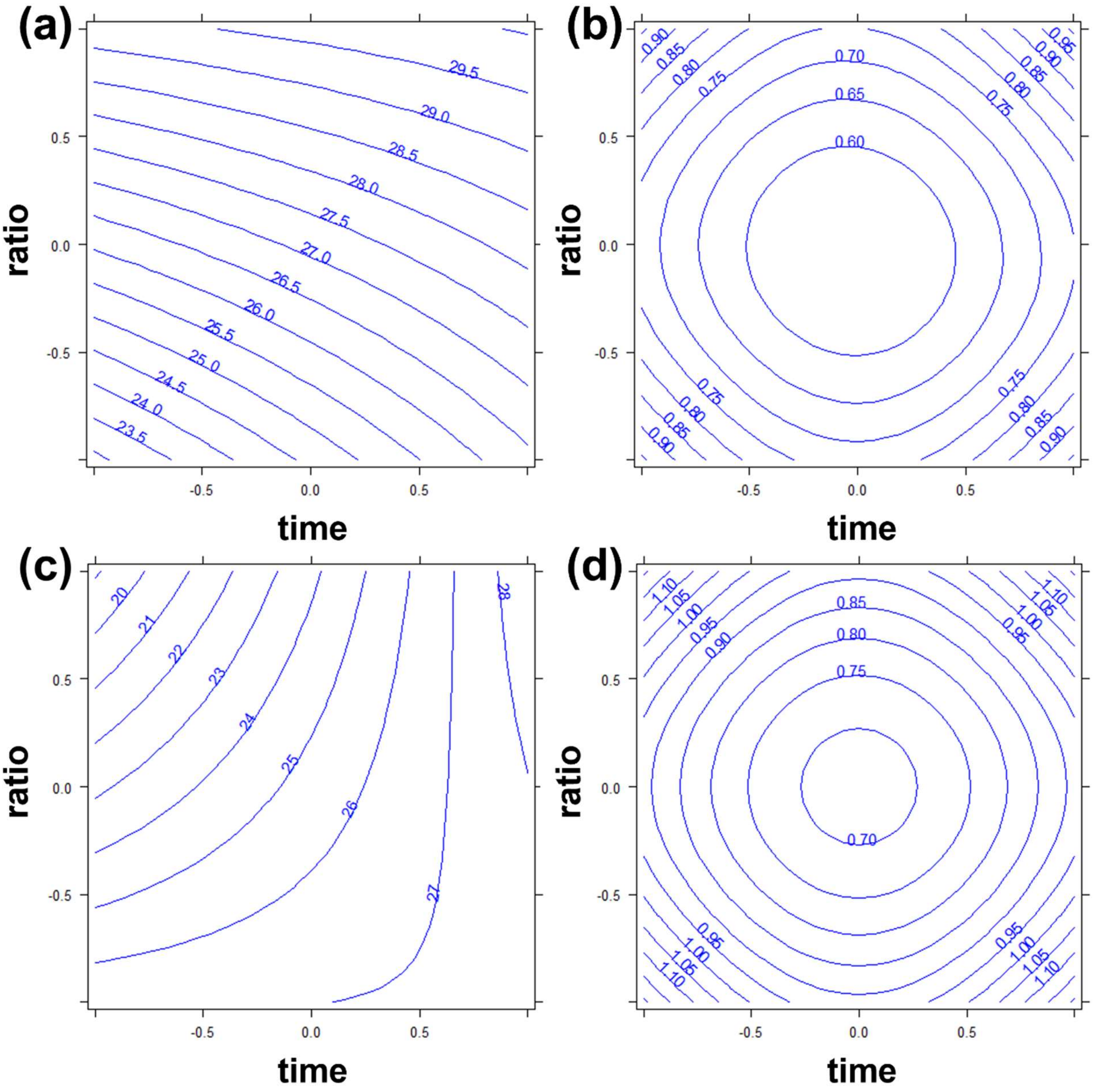

Supplementary Figure S3. (a,c) Response surface and (b,d) semiamplitude of the 95% confidence interval for (a,b) red Merlot GP and (c,d) white Garganega GP models. Factors time and ratio are reported in abscissa and ordinate respectively. Enzyme type was kept to Driselase level. The axis scale ±1 is due to DoE levels.

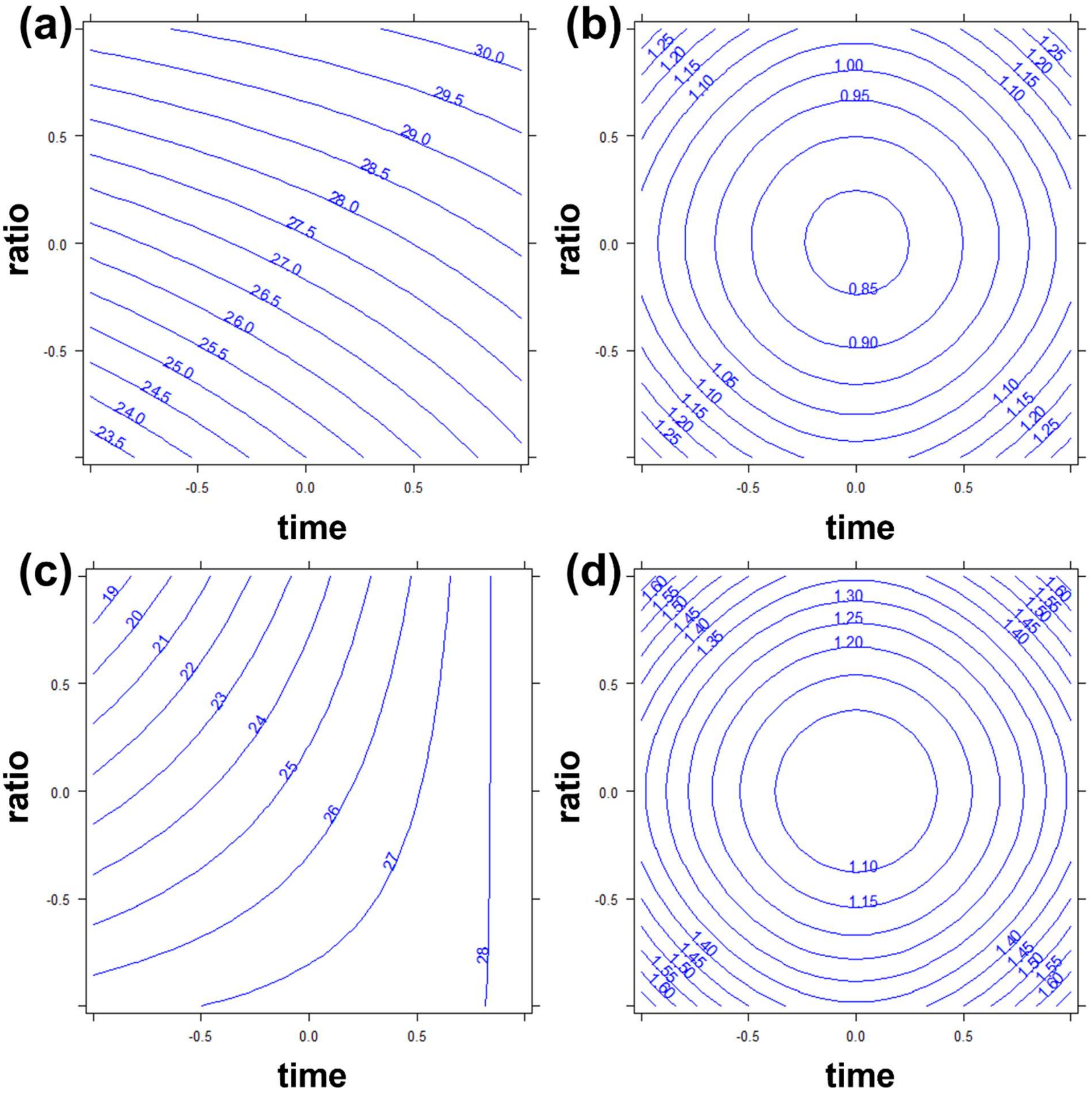

Supplementary Figure S4. (a,b) Response surface and (b,d) semiamplitude of the 95% confidence interval for (a,b) red Merlot GP and (c,d) white Garganega GP models. Factors time and ratio are reported in abscissa and ordinate respectively. Enzyme type was kept to Viscozyme level. The axis scale ±1 is due to DoE levels.
